# Dr. Schœpf Merei

**Published:** 1858-05

**Authors:** 


THE eminent Hungarian physician, Dr. SCHŒPF MEREI, died on the 12th of March, in the fifty-fourth year of his age. He was Professor of the History of Medicine in the University of Pesth, and the founder of the Children's Hospital of that city; one of the first institutions of its kind in Europe. He enjoyed a large practice, and was regarded as one of the first medical men in Hungary. He was possessed of varied and extensive literary acquirements, and spoke with great fluency many of the languages of Europe.

